# Patterns of Dementia and Serious Mental Illness Co-occurrence among Nursing Home Residents: A Latent Class Analysis

**DOI:** 10.1093/geroni/igaf122.3822

**Published:** 2025-12-31

**Authors:** Laura Block, Roger Brown, Donovan Maust, Megan Zuelsdorff, Andrea Gilmore-Bykovskyi, Tonya Roberts

**Affiliations:** University of Wisconsin-Madison, Madison, Wisconsin, United States; University of Wisconsin-Madison, Madison, Wisconsin, United States; University of Michigan Medical School, Ann Arbor, Michigan, United States; University of Wisconsin Madison, Madison, Wisconsin, United States; University of Wisconsin-Madison School of Medicine and Public Health, Madison, Wisconsin, United States; University of Wisconsin-Madison, Madison, Wisconsin, United States

## Abstract

Over half of all nursing home (NH) residents live with Alzheimer’s disease and Alzheimer’s disease related dementias (AD/ADRD), and a third with serious mental illness (SMI). AD/ADRD and SMI frequently co-occur among older adults, with research suggesting co-occurrence associates with heightened symptom burden and poor outcomes. Despite high rates of AD/ADRD and SMI among NH residents, patterns of AD/ADRD and SMI co-occurrence among this population and related resident-level characteristics have not been robustly examined. This study conducted a cross-sectional analysis of NH resident quarterly assessment data from the Minimum Data Set (MDS) 3.0 FY2019, utilizing unconditional latent class analysis (LCA) to examine patterns of AD/ADRD and SMI co-occurrence. Among a sample of 227,651 residents with AD/ADRD and/or SMI, a six-class model provided the best-fitting, clinically relevant solution. Classes were labeled according to most probable diagnoses: Predominantly Depression (n = 74,298, 32.6%), Predominantly Dementia (n = 53,667, 23.6%), Anxiety-Depression-Dementia, (n = 68,854, 30.2%) Mixed Diagnoses with Dementia (n = 12,945, 5.7%), Mixed Diagnoses (n = 13,517; 5.9%), and Predominantly Schizophrenia (n = 4,370, 1.9%). Classes differed significantly across clinical characteristics; for example, residents in the Anxiety-Depression-Dementia class had higher rates of pain while residents in the Mixed Diagnoses with Dementia, Mixed Diagnoses, and Predominantly Schizophrenia classes had the highest rates of behavioral symptoms. A more nuanced understanding of AD/ADRD and SMI co-occurrence among NH residents and class differences suggest diagnostic case-mix and resident-level care needs can together inform resource allocation and care delivery. Future work incorporating additional data sources and longitudinal approaches to explore relationships between symptoms, care measures, and outcomes is merited.

